# Social Network Plasticity of Mice Parental Behavior

**DOI:** 10.3389/fnins.2022.882850

**Published:** 2022-06-07

**Authors:** Chitose Orikasa

**Affiliations:** Laboratory for Morphological and Biomolecular Imaging, Nippon Medical School, Tokyo, Japan

**Keywords:** melanin-concentrating hormone, oxytocin, maternal behavior, neural plasticity, social isolation, *γ*-aminobutyric acid

## Abstract

Neural plasticity occurs during developmental stages and is essential for sexual differentiation of the brain and the ensuing sex-dependent behavioral changes in adults. Maternal behavior is primarily affected by sex-related differences in the brain; however, chronic social isolation even in mature male mice can induce maternal retrieving and crouching behavior when they are first exposed to pups. Social milieus influence the inherent behavior of adults and alter the molecular architecture in the brain, thereby allowing higher levels of associated gene expression and molecular activity. This review explores the possibility that although the development of neural circuits is closely associated with maternal behavior, the brain can still retain its neuroplasticity in adults from a neuromolecular perspective. In addition, neuronal machinery such as neurotransmitters and neuropeptides might influence sociobehavioral changes. This review also discusses that the neural circuits regulating behaviors such as parenting and infanticide (including neglect behavior), might be controlled by neural relay on melanin concentrating hormone (MCH)–oxytocin in the hypothalamus during the positive and negative mode of action in maternal behavior. Furthermore, MCH–oxytocin neural relay might contribute to the anxiolytic effect on maternal behavior, which is involved with reward circuits.

## Introduction

**FIGURE 1:**
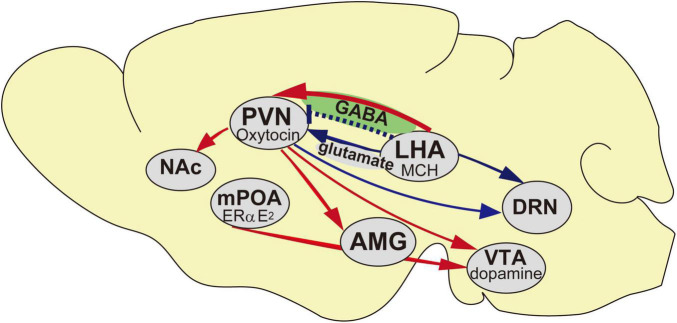
Schematic representation of the neural networks of the maternal behavior in mice. Blue arrows indicate inhibitory inputs to trigger pup avoidance, red arrows indicate neural networks in accelerating maternal behavior and blue dotted line indicates aggressiveness toward pups if it is lacking at the embryonic day. AMG, Amygdala; DRN, Dorsal raphe nucleus; LHA, Lateral hypothalamic area; mPOA, Medial preoptic area; NAc, Nucleus Accumbens; PVN, Paraventricular nucleus; VTA, Ventral tegmental area.

**FIGURE 2:**
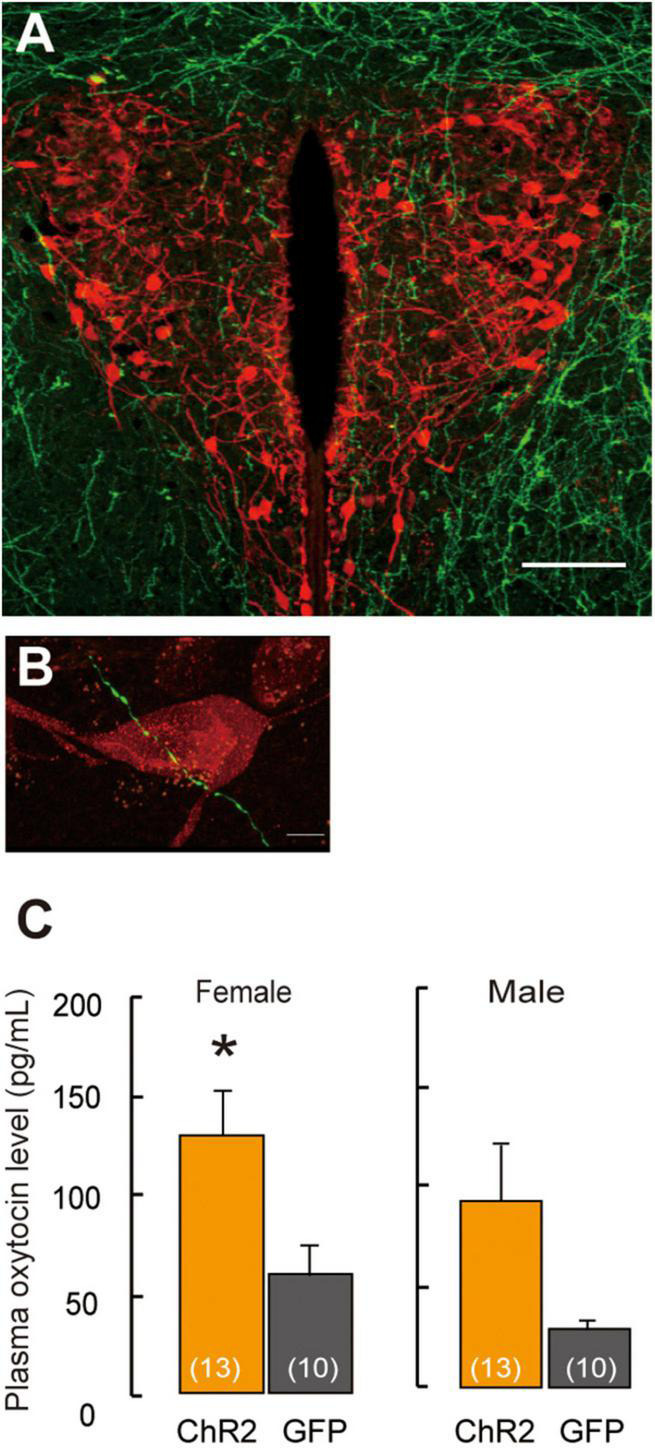
Projection of ChR2-EYFP fibers form LHA-MCH neurons into PVN-oxytocin neurons. **(A)** ChR2-EYFP fiber (green) from MCH neurons surroundings and into the PVN. Scale bar, 100 μm. **(B)** ChR2-EYFP fiber (green) identified in closely vicinity to the oxytocin neurons (red). Scale bars, 5 μm. **(C)** Oxytocin levels in plasma samples by the stimulation of optogenetics in the PVN. Mean ± SEM *t*-test, *p < 0.05 [modified from Kato et al. (2021)].

Neural plasticity is critical for sex-dependent changes responsible for the neural circuit of the brain and alterations in various aspects of adult sex related behavior (McEwen et al., 1977; MacLusky and Naftolin, 1981; MacLusky et al., 1985) that is restricted to a particularly critical period during development. Differences in reproductive behavior including courtship, copulatory, and parental behaviors, between the sexes are attributable to the different neural circuit mechanisms. However, in adult male mice, chronic social isolation induces maternal retrieving and crouching behavior when they are first exposed to pups (Orikasa et al., 2015). We detected differences in the expression of the melanin-concentrating hormone (*MCH*) gene in the hypothalamus between isolated males exhibiting parental behavior and group-housed males ignoring pups using microarrays (unpublished data). MCH neurons are responsible for the changes that are involved in maternal behavior in male mice, and it has become possible to modulate *MCH* expression *via* social stimuli. MCH receptor (MCHR) 1 is involved in the stress response (Smith et al., 2006). Social stress also results in alterations in the endocrine system, such as changes in the levels of glucocorticoids, oxytocin, and vasopressin (Mumtaz et al., 2018). Moreover, the hormonal milieu, such as estrogen and progesterone levels in the plasma, drastically affects the resulting stimulus lactation (Voogt, 1978), which is possible because of the alternating gene expression in the brain. In the medial preoptic area (mPOA), MCH neurons colocalized with estrogen receptor (ER)α during the transient expression of late lactation to weaning (Teixeira et al., 2020), which is possibly involved in the regulation of reproductive maternal behavior in the brain. The higher levels of MCH in lactating mice colocalize with ERα and the signal transducer and activator of transcription 5 (STAT5) in the mPOA (Teixeira et al., 2020). MCH is a neuropeptide secreted from the lateral hypothalamic area (LHA; Bittencourt and Elias, 1998; Sita et al., 2007), with distinguishing physiological features such as energy homeostasis (Qu et al., 1996; Rossi et al., 1997; Shimada et al., 1998; Marsh et al., 2002). The other roles of MCH neurons in neurophysiology are regulating sleep (Tsunematsu et al., 2014; Komagata et al., 2019), stress (Oh et al., 2020), cognition (Sherwood et al., 2012), memory (Izawa et al., 2019), and anxiety (Concetti et al., 2020). As such, MCH acts as a neuromodulator that integrates various physiological functions. Recent evidence revealed a neural relay of MCH with the neurotransmitter γ-aminobutyric acid (GABA) projecting to the paraventricular nucleus (PVN) oxytocin neurons (Kato et al., 2021; Figures 1, 2A,B). In this case, no sex difference in maternal behavior was observed when they were both pharmacogenetically and optogenetically stimulated. Galanin neurons in the mPOA played a role in mouse maternal behavior in both sexes, i.e., failure of parental response after genetic ablation of mPOA galanin neurons (Wu et al., 2014). These results provide information on the male brain at a circuit level including the regulation of parental behavior.

This review proposes that social stimuli can affect social behavior including the positive and negative balance of the behavior assembly using neuropetides and neurotransmitters in the hypothalamus of mice.

## Sex Difference and Implications of the Medial Preoptic Area in Parental Behavior

The mPOA plays a pivotal role in maintaining the neural circuit involved in social behavior, which regulates male copulatory behavior and maternal behavior in rodents (Tachikawa et al., 2013; Numan, 2020). The mPOA is a sexual dimorphic region of the brain that expresses gonadal steroid hormone receptors such as estrogen (DonCarlos et al., 1995; Yokosuka et al., 1997; Orikasa et al., 2002) and androgen receptors (Portillo et al., 2006). Estrogen results in behavioral actions females and prompts maternal care through the ERα in the mPOA (Ribeiro et al., 2012; Fang et al., 2018). When estradiol affects mPOA, it results in an increased expression of ERα in pregnancy, thereby inducing maternal behavior (Numan, 1988). However, systemic estradiol administration decreased maternal retrieving and licking and nursing behaviors (Kasper and Telegdy, 1975). Excessive estradiol in adults induces aggressiveness toward pups (Fukui et al., 2022). Furthermore, decreased maternal behavior due to estradiol administration implied that the ERα mRNA expression was downregulated in the mPOA and medial amygdala (mAMY; Murakami, 2016). Moreover, ERα in the mPOA is downregulated by the neonatal sex steroid in males, i.e., testosterone, and its enzymatic derivative, estradiol. This is the organizational effect of sex difference that leads to decreased ERα expression in males compared with females (Orikasa et al., 1996; Yokosuka et al., 1997). Therefore, ERα expression is much higher in females than in males, assuring higher expression in adulthood. Neonatal exposure to xenoestrogens, such as bisphenol A, leads to impaired maternal behavior in adults (Palanza et al., 2002; Monje et al., 2007). Female ERα knockout rats show impaired maternal behavior (Leckman and Herman, 2002). These results suggest that the involvement of ERα expression in maternal behavior is determined by neonatal sex steroids. However, optogenetical stimulation of the ERα neurons in the mPOA induces mounting and pup retrieval behavior, regardless of sex (Wei et al., 2018). Therefore, females also have the neural circuit that is responsible for the typical male behavior in the brain.

## Social Cues Alter Behavioral Changes and Endocrine Systems

Males that received social stimuli, such as cohabitation, and experienced delivery with pregnant females exhibited behavioral changes, showing maternal behavior (Tachikawa et al., 2013). Social experiences may modulate the changes in behavior responses. An environmental cue, such as social experience in adults, may affect the plasticity of the neurobiological pathways that govern the responsiveness to cognition and reproduction in adults. Social stressors, such as social isolation, affect reproduction (Orikasa et al., 2015), recognition memory (Leser and Wagner, 2015), depression (Grippo et al., 2007), and social behavior (Tan et al., 2019) as well as trigger anxiety-like behavior (Kumari et al., 2016; Donovan et al., 2020) and aggression (Oliveira et al., 2019; Tan et al., 2019). Social isolation has been known to instigate considerable stress in rodents, leading the animal to show indications of prosocial behavior. Exclusive social isolation causes reversible dysfunction in the social recognition memory (Leser and Wagner, 2015) by impairing the glutamatergic signaling between the olfactory bulb and dorsal hippocampus (HPC; Almeida-Santos et al., 2019). During chronic social isolation for prolonged periods of time, arginine vasopressin mRNA and corticosterone are seen to be elevated (Heck et al., 2020). The transcriptional machinery in the genome interacts with hormones and hormone receptors, which in turn lead to alternation in several behavioral traits. Social stress in adults can also alter neural plasticity, resulting in behavioral changes.

## Social Cues Alter Behavioral Changes at the Cellular and Molecular Levels

How do social stimuli change behavior? Social cues acting on the nervous system can change the brain morphology and physiological properties. A previous study showed that in the posterodorsal mAMY, the neuronal volume in the nucleus can increase after testosterone exposure in adulthood (Cooke et al., 1999). Testosterone also affects the soma size of the spinal nucleus of the bulbocavernosus (Forger et al., 1992). Synaptic reorganization in the arcuate nucleus of rats is dependent on the effect of estradiol in adults (Csakvari et al., 2008) and the somatic volume of the posteroventral mAMY during the estrous cycle (RochaI, Mestriner et al., 2007). In the HPC, external environmental conditions influence the activity and plasticity of the dentate gyrus in adult and prepubertal male rats. In addition to sex-dependent differences in the HPC activity, effects of the prenatal stress conditions are observed in the adult HPC (Mychasiuk et al., 2016). Neural plasticity in the sexually dimorphic nucleus involved in the cognitive and reproductive functions is evident in mature individuals. However, the underlying cellular and molecular mechanisms of the implications of environmental signals on alternations in brain function and behavior need to be considered.

Previous reports have documented that experience-dependent modifications of chromatin structure and transcriptional regulation can affect neural plasticity in the adult visual cortex (Maya-Vetencourt et al., 2012; Baroncelli et al., 2016). Histone posttranslational modifications and DNA methylation affect the synaptic connectivity in the adult HPC, resulting in regulated maternal care (Bagot et al., 2012). Nucleosomal histone turnover and chromatin remodeling contribute to changes in gene expression and synapse formation and these presumably arise owing to behavioral changes (Maze et al., 2015). DNA modifications and neurogenesis in the HPC may be implicated in neuronal plasticity in the structure of the adult brain (Ma et al., 2009). These labile phenomena involve a complex interplay of cellular events in the central nervous system and allow neural plasticity adaptations. ERα is associated with the induction of oxytocin receptor (OXTR) binding in the mPOA (Young et al., 1998) and regulation of maternal behavior (Champagne et al., 2006). Alternatively, DNA methylation of the OXTR changed the licking–grooming levels of rat dams (Danoff et al., 2021) in neonates, thereby affecting maternal behavior sustained in adulthood. DNA methylation in the promoter region of ERα, where the methylation patterns are associated with maternal care received during infancy, results in changes in gene expression in the mPOA of the offspring, thus affecting maternal behavior (Champagne and Curley, 2008).

## Melanin Concentrating Hormone–Oxytocin Circuit Architecture in Maternal Behavior

Disruption of the neural relay of the MCH–oxytocin signaling, as seen in the case of MCH knockout from embryonic development with the Tet-off system (Kato et al., 2021), might alter the efficacy of oxytocin anxiolytic buffering, resulting in an infanticidal tendency in males. Ablating MCH neurons in *MCH-tTA; TetO DTA* bigenic +/+ virgin males make them more aggressive toward pups, whereas bigenic +/+ virgin females are apathetic toward pups, similar to +/+ bigenic mothers (Kato et al., 2021). The ablation of MCH neurons affects the body weight, which appears to be decreased in both sexes (Kato et al., 2021), and this finding is consistent with the results of a previous report showing engagement in infanticide due to hyperactivity (Kokkotou et al., 2005; Zhou et al., 2005; Chee et al., 2019). A study using Cre-dependent DTA systems reported that adults lacking approximately 70% of MCH-expressing neurons spent less time exhibiting maternal behavior, whereas no infanticide was observed in males (Kato et al., 2021). Thus, the absence of MCH neurons induced adults to perform infanticide and ignore pups. However, the ablation of MCH neurons, specifically at the embryonic stage, in males has a profound effect on aggressiveness toward pups because of the possible impairment of establishing the neural relay on MCH and oxytocin.

Melanin concentrating hormone neurons are located in the LHA and project extraordinarily throughout the brain, acting as neuromodulators and orchestrating physiological functions. MCHRs are also distributed in the reward circuits, nucleus accumbens (NAc), PVN, and central amygdala in the brain. MCHR1 and MCHR2 have been identified and these mediate physiological functions, such as the regulation of stress response in rodents. The administration of MCH antagonists (Shimazaki et al., 2006) and occurrence of null mutations in *MCHR1* result in anxiolytic-like behavior (Roy et al., 2006). Therefore, this effect of MCH neurons leads to the induction of social liability stress and anxiety.

## Oxytocin Involved in Maternal Behavior

MCH receptors on the oxytocin neurons in the PVN are scanty; therefore, the MCH neurotransmitter glutamate or GABA mainly regulates oxytocin dependent neural activity. Social isolation under experimental conditions is supposed to affect specific attributes profoundly. The neural relay of MCH–oxytocin signaling might result in the emotional reinforcement of reward processing. Oxytocin has been suggested to be involved in social reward and buffering (Kikusui et al., 2006). While oxytocin may activate the dopaminergic reward pathways in response to social cues (Strathearn, 2011), an ERα knockout disrupts maternal behavior (Ribeiro et al., 2012) and increases infanticide (Champagne and Curley, 2008) caused by aberrant oxytocin (Young et al., 1998) and dopaminergic signaling (Küppers et al., 2008). Oxytocin has been known to play a role in relieving anxiety, inducing social bonding and decreasing social stress in prairie voles (Smith and Wang, 2014). It is also involved in the associated refinement of stress, which is concomitant with social buffering (Kikusui et al., 2006; Burkett et al., 2016). Elevation in oxytocin levels synchronously coincides with that of corticosterone levels, indicating the antagonism of a negative and positive balance in the mode of behavioral action (Burkett et al., 2016). High corticosterone levels in the plasma are associated with a negative mode of behavior action, leading to depression (Skórzewska et al., 2019). In contrast, high oxytocin levels in the plasma indicate a positive mode of behavior action, resulting in the exhibition of prosocial and anxiolytic effects (Onaka et al., 2012). Whether the brain is responsible for behaviors including a negative balance, especially in the case of infanticide, has not been well clarified. For instance, oxytocin antagonist and dopamine D1 receptor blockers in the ventral tegmental area (VTA) result in impaired maternal behavior (Strathearn, 2011); therefore, oxytocin neurons projecting from both the mPOA and PVN to the VTA are involved in this behavior in rats (Shahrokh et al., 2010; Figure 1). Moreover, oxytocin is responsible for social bonding in prairie voles (Walum and Young, 2018). Photostimulation of ChR2 fiber in the PVN coincides with pup presentation resulting in higher oxytocin levels in plasma samples measured in females (Kato et al., 2021), whereas in males, there is a higher propensity for oxytocin levels in ChR2 (Figure 2C). However, the mechanisms underlying the activity of oxytocin neurons under chronically stressed conditions remain controversial. Longer periods of isolation affect the possible conformational changes in the brain structure and neural plasticity, as was evident from an experiment wherein a social isolation period of 1 week was inadequate for integrating maternal behavior, whereas that of 3 weeks notably resulted in retrieving and crouching behavior in male mice (Orikasa et al., 2015). An isolation period of 4 weeks also increased plasma oxytocin levels (Grippo et al., 2007), indicating that the length of the isolation period affects plasma oxytocin levels (Grippo et al., 2007; Kato et al., 2021). Moreover, moderate stress, such as acute 3 h immobilization, can also increase oxytocin signaling (Muroy et al., 2016). Thus, variations in stress conditions depending on physical or psychological stimuli on the animal, such as electric shock to the feet, chronic restraint stress or conditioned fear, and stress-evoking fear, might influence oxytocin activation and increase plasma oxytocin levels (Onaka, 2004).

## Involvement of *γ*-Aminobutyric Acid in Maternal Behavior

Projection of MCH neurons to oxytocin neurons in the PVN has been shown in a recent study (Kato et al., 2021; Figure 2A), thus revealing the neural relay from LHA–MCH to the PVN–oxytocin circuits that are involved in the maternal behavior of females and males. The fibers of MCH neurons, which were labeled fluorescently by ChR2–yellow fluorescent protein (EYFP), were detected in the oxytocin neurons in the PVN. Excitatory GABAergic innervation derived from MCH neurons (Figure 2B) might regulate the PVN-oxytocin neurons, as the application of the GABA agonist muscimol *via* infusion in the vicinity of the PVN, resulted in increased *c-fos* expression in oxytocin neurons in social isolation compared with group-housing in both sexes (Kato et al., 2021). Therefore, social isolation stress may change MCH GABAergic innervation into oxytocin as an excitatory pathway. A previous study has shown that GABA changes to an excitatory pathway during development (Rheims et al., 2008) and even in adults (Gies and Theodosis, 1994; Marty and Llano, 2005; Kim et al., 2011; Lee et al., 2015; Choi et al., 2016). In the case of maternal behavior, GABAergic innervation in the mPOA regulates anxiolytic effects on mood and emotion (Zhang et al., 2021) because lesions of this area have been shown to interfere with maternal behavior (Fleming et al., 1983). GABAergic neurons in the mPOA exert a positive, anxiolytic effect and facilitate parental behavior (Zhang et al., 2021).

## Positive and Negative Balance of Parenting and Infanticide/Ignore

Maternal behavior involves direct interaction with social stimuli from infants, either positive or negative, such as maternal care versus apathetic behavior or aggression toward pups. How are the neural circuits involved in these opposing behavioral traits controlled? In the mAMY, the activity of GABA induces changes in parenting behavior against infanticide in males, whereas no changes in the actions that are consistent with the GABAergic neural activity are observed in females (Chen et al., 2019). The upregulation of OXTR in the accessory olfactory bulb and ventromedial hypothalamus, together with its downregulation in the lateral septum and anterior olfactory area might be responsible for the tendency of committing infanticide (Olazábal and Alsina-Llanes, 2016). The pup pheromonal signals that are detected in sexually naïve male mice in the vomeronasal organ (VNO) induce infanticide (Tachikawa et al., 2013). In contrast, the surgical removal of the VNO in sexually naïve male mice or in males that underwent mating and who cohabitation with pregnant females suppresses infanticide (Tachikawa et al., 2013). Therefore, sexually naïve male mice or mating males who cohabit with pregnant females alter parental behavior by first blocking chemosensory signals that are transferred into the vomeronasal sensory neurons (Tachikawa et al., 2013). In our previous study, we identified another neural circuit involved in infanticide using a conditional knockout model created using the Tet-off system, MCH-knockout male mice exhibited a tendency to commit infanticide (Kato et al., 2021). Male mice lacking MCH display a phenotype of increased locomotor activity (Kokkotou et al., 2005; Zhou et al., 2005). In terms of specific behavior, the ablation of MCH neurons in males results in increased aggressiveness toward pups and intruder male mice, whereas females exhibit a lack of care and tend to ignore the pups (Kato et al., 2021). Therefore, the effect of ablating MCH neurons is appreciably diverse between sexes. The lack of MCH neurons is suggested to affect sex-related differences in maternal behavior. In mice, when MCH neurons are counteracted in the brain area involved in that specific behavior, leading to disinhibition of the neural network owing to aggressiveness by neuronal ablation, mice are destined to infanticidal tendency. A recent paper has shown that male mice with increased in GABA expression in mAMY exhibit significantly higher infanticidal tendency (Chen et al., 2019) as GABA plays a role in innate behaviors and is activated by the neural networks of pheromonal signals from sensory organs (Swanson, 2003; Hong et al., 2014; Unger et al., 2015). The sex-related differences observed in the mAMY are associated with maternal behavior (Chen et al., 2019). In other words, females display pup grooming (Chen et al., 2019) in contrast to the observations of the study regarding the inhibition of the maternal behavior in rodents with lesions in the mAMY (Sheehan et al., 2001). Subcutaneous administration of oxytocin inhibits infanticide in female mice (McCarthy, 1990). Oxytocin neurons in the hypothalamus innervate to the amygdala nucleus to regulate GABAergic interneurons, resulting in decreased aggressiveness (Knobloch et al., 2012). GABA and glutamatergic neurons in the mPOA regulate opposing behavior and anxiety-like and affiliative parental behavior, respectively (Zhang et al., 2021). Glutamatergic neurons are responsible for anxiogenic effects, whereas GABAergic neurons produce anxiolytic effects on the parental behavior. MCH neurons express GABA (Kato et al., 2021) and glutamate (Chee et al., 2015), which possibly influence maternal behavior and aggressiveness toward pups, respectively. Alternatively, abolishing oxytocinergic projection can consequently raise the antithetic behavior response to maternal behavior, such as overt aggressiveness and is an inherent attribute of the amygdala. Urocortin 3 (Ucn3) in the perifrontal area (PFA), which is distributed between the fornix and PVN that is activated during the course of committing infanticide, is another related neural circuit. However, the Ucn3 (Autry et al., 2021) neurons in the PFA exhibit their presumptive negative role in parenting.

Although optogenetic studies have demonstrated certain functional properties of some of the maternal behavior traits in PVN–oxytocin neurons and infanticide in PFA–Ucn3 neurons, little is known about the mechanisms of cooperation between oxytocin and Ucn3 neurons and the interactions in the switch from maternal behavior to committing infanticide. Whether abolishing the neural relay from the LHA–MCH to the PVN–oxytocin affects the PFA–Ucn3 neurons and results in pup-direct aggression is a point that remains to be elucidated. On one hand, OXTRs expressed in the serotonergic dorsal raphe nucleus (DRN; Grieb and Lonstein, 2021) are possibly involved with particular reference to ignore pups in the midbrain site. On the other hand, MCH innervated to the DRN serotonergic neurons (Devera et al., 2015; Figure 1) might be involved in this behavior. Nevertheless, whether or not the definitive cardinal site of each infanticidal and neglect behavior is the same, the possible underlying cause of each behavior is in the MCH. The neural relay from the LHA–MCH to the PVN–oxytocin might contribute to the anxiolytic effect, including oxytocin that disinhibits the nucleus, thereby governing agonistic behavior. Null study for oxytocin and its receptor is thought be related to maternal behavior (Bridges, 2015). Oxytocin terminates the VTA that is involved in the prosocial (Hung et al., 2017; Figure 1) and maternal behavior (Shahrokh et al., 2010; Strathearn, 2011), and maternal or social bonding involved in the regulation of NAc dopamine levels (Shahrokh et al., 2010). MCHRs are also distributed in the reward circuit. The NAc is involved in maternal behavior (Alachkar et al., 2016). Moreover, the dopaminergic neurons in the VTA are activated by the indirect activation of ERα in the mPOA (Figure 1) when inducing maternal behavior (Fang et al., 2018).

## Neural Circuit Associated With Difference in Parenting Between Virgin Males and Fathers

Male mice exhibit lesser infanticide after going through mating, gestation, childbirth with females (vom Saal and Howard, 1982; Tachikawa et al., 2013). Male brain circuit might drastically change after social encounter with females. The neural circuit responsible for parenting in fathers and socially isolated males might have similar characteristics. Virgin males in which the vomeronasal neural pathway is activated can induce infanticide, whereas those in which the VNO is removed or fathers cohabiting with females exhibit alloparental behavior (Tachikawa et al., 2013). The switch from parental to infanticidal behavior is due to the balance of neural activation between the preoptic area and bed nucleus of the stria terminalis (Tsuneoka et al., 2015). In our previous study, VNO-specific ion channel knockout Trpc2^–/–^ was shown to accelerate parental behavior in sexually naïve male mice after social isolation (Orikasa et al., 2017). Therefore, socially isolated virgin and sexually experienced male mice cohabiting with pregnant females display similar VNO characteristics in terms of neural circuit. Isolated males are introduced to parenting *via* first time encounter with pups by means of activation of innate neural circuit, and this behavior could be exhibited in the absence of prior experiences. On the contrary, parental behavior of habituated mating male mice might be elicited by social experience rather than owing to innate characteristics (Tsuneoka et al., 2015). The neural circuit associated with parenting between virgin males and fathers remains to be elucidated.

## Discussion

Sexual dimorphism in the brain related to behavior occurs primarily due to the genotype as well as perinatal hormones that are secreted during sensitive periods of development. Environmental cues such as social signals could change the plasticity of the neural circuits in maternal behavior, even in adults. Social isolation positively changes the behavioral action depending on the possible innate neural circuit activation even after sexual dimorphism has occurred in the brain. In this review, we have discussed the genetic control mechanisms underlying the changes relevant to parental behavior. Switching of the positive and negative mode of behavioral action, i.e., between parenting and aggressiveness toward pups (including less concerned pups) has also been discussed. Consequential disruption of neural relay on MCH–oxytocin signaling by MCH knockout specifically by passing through in the Tet-off system may result in the loss of oxytocin buffering system organization, subsequently resulting in the infanticide of pups. In addition, GABA is the potential candidate responsible for the occurrence of two modes of behavioral action. Neural relay on MCH–oxytocin may contribute to the anxiolytic effect on parental behavior, thereby contributing reward circuits. In recent neuroscientific research, optogenetic studies are the mainstream and the potential tool to improve the understanding the brain function; however, these studies should be performed on physiologically valuable approach.

## Author Contributions

The author confirms being the sole contributor of this work and has approved it for publication.

## Conflict of Interest

The author declares that the research was conducted in the absence of any commercial or financial relationships that could be construed as a potential conflict of interest.

## Publisher’s Note

All claims expressed in this article are solely those of the authors and do not necessarily represent those of their affiliated organizations, or those of the publisher, the editors and the reviewers. Any product that may be evaluated in this article, or claim that may be made by its manufacturer, is not guaranteed or endorsed by the publisher.
